# Diagnostic Bacteriology in District Hospitals in Sub-Saharan Africa: At the Forefront of the Containment of Antimicrobial Resistance

**DOI:** 10.3389/fmed.2019.00205

**Published:** 2019-09-23

**Authors:** Jan Jacobs, Liselotte Hardy, Makeda Semret, Octavie Lunguya, Thong Phe, Dissou Affolabi, Cedric Yansouni, Olivier Vandenberg

**Affiliations:** ^1^Department of Clinical Sciences, Institute of Tropical Medicine Antwerp, Antwerp, Belgium; ^2^Department of Microbiology and Immunology, KU Leuven, Leuven, Belgium; ^3^JD MacLean Centre for Tropical Diseases, McGill University, Montreal, QC, Canada; ^4^Department of Clinical Microbiology, National Institute of Biomedical Research, Kinshasa, Democratic Republic of Congo; ^5^Service of Microbiology, Kinshasa General Hospital, Kinshasa, Democratic Republic of Congo; ^6^Sihanouk Hospital Center of HOPE, Phnom Penh, Cambodia; ^7^Clinical Microbiology, Centre National Hospitalier et Universitaire Hubert Koutoukou MAGA, Cotonou, Benin; ^8^Center for Environmental Health and Occupational Health, School of Public Health, Université Libre de Bruxelles (ULB), Brussels, Belgium; ^9^Innovation and Business Development Unit, LHUB - ULB, Pôle Hospitalier Universitaire de Bruxelles (PHUB), Université Libre de Bruxelles (ULB), Brussels, Belgium; ^10^Division of Infection and Immunity, Faculty of Medical Sciences, University College London, London, United Kingdom

**Keywords:** antimicrobial resistance (AMR), antimicrobial stewardship (AMS), infection prevention and control (IPC), low-resource settings (LRS), clinical and bacteriology, Sub-Saharan Africa

## Abstract

This review provides an update on the factors fuelling antimicrobial resistance and shows the impact of these factors in low-resource settings. We detail the challenges and barriers to integrating clinical bacteriology in hospitals in low-resource settings, as well as the opportunities provided by the recent capacity building efforts of national laboratory networks focused on vertical single-disease programmes. The programmes for HIV, tuberculosis and malaria have considerably improved laboratory medicine in Sub-Saharan Africa, paving the way for clinical bacteriology. Furthermore, special attention is paid to topics that are less familiar to the general medical community, such as the crucial role of regulatory frameworks for diagnostics and the educational profile required for a productive laboratory workforce in low-resource settings. Traditionally, clinical bacteriology laboratories have been a part of higher levels of care, and, as a result, they were poorly linked to clinical practices and thus underused. By establishing and consolidating clinical bacteriology laboratories at the hospital referral level in low-resource settings, routine patient care data can be collected for surveillance, antibiotic stewardship and infection prevention and control. Together, these activities form a synergistic tripartite effort at the frontline of the emergence and spread of multi-drug resistant bacteria. If challenges related to staff, funding, scale, and the specific nature of clinical bacteriology are prioritized, a major leap forward in the containment of antimicrobial resistance can be achieved. The mobilization of resources coordinated by national laboratory plans and interventions tailored by a good understanding of the hospital microcosm will be crucial to success, and further contributions will be made by market interventions and business models for diagnostic laboratories. The future clinical bacteriology laboratory in a low-resource setting will not be an “entry-level version” of its counterparts in high-resource settings, but a purpose-built, well-conceived, cost-effective and efficient diagnostic facility at the forefront of antimicrobial resistance containment.

## Main Terms, Definitions, and Abbreviations Used in This Review

In this review, we use the term “antimicrobial resistance” (AMR), which is the ability of a microorganism to resist antimicrobial treatment. AMR encompasses antibiotic resistance and the ability of bacteria to resist antibiotic treatment in both human and animal healthcare.

The review focuses on “low-resource settings” (LRS), which include low-income countries as well as remote, rural and underserved areas in middle-income countries. We would like to note that we incidentally also use the term “low- and middle-income countries” (LMIC^1^) when it is used in an original reference cited. We mainly discuss Sub-Saharan Africa because most low-income countries (27 of 34) (1) are located there, and recent initiatives have spurred laboratory-related capacity building in the region.

The term “One Health,” which is often used in this review, refers to a multisectoral, multilevel and transdisciplinary approach aiming to achieve optimal health outcomes by recognizing the interactions between humans, animals, and the environment.

The review focuses on the “clinical bacteriology laboratory” (CBL). However, given the scarcity of literature about the CBL in low-resource settings, we present complementary data from general (human) diagnostic laboratories, termed “medical laboratories.” These medical laboratories in referral or district hospitals provide hematology, biochemistry, microscopy (tuberculosis and parasites) and blood transfusion services and are staffed by laboratory technicians with broad and basic training, who are commonly referred to as “laboratory staff.”

## Antimicrobial Resistance is a Public Health Treat Worldwide, But Low- and Middle-Income Countries are Hit Hardest

*Antibiotic consumption, in human and animal health as well as in agriculture, is constantly increasing. As a result, antimicrobial resistance rates are rising worldwide. To tackle this, a “One Health” approach is needed. LMIC are hit hardest by AMR. This is partly due to budgetary problems but also because of social norms and beliefs that impede the proper diagnosis and treatment of health problems in patients*.

### Antimicrobial Resistance Has Recently Been Accelerated by Human Factors in Different Sectors

Antibiotics have revolutionized the treatment of infectious diseases. Therefore, antibiotics are currently being used in nearly every facet of modern medicine in domains such as organ transplantation, invasive surgery and cancer treatment (2, 3). However, after the introduction of nearly every new antibiotic product or class, AMR has emerged (4).

In the first 10 years of this century, human antibiotic consumption increased by 35%, and AMR rates have risen, particularly in countries with high antibiotic consumption (5)^4^. Antibiotic consumption is not restricted to humans but also occurs in animal health and agriculture, making it a “One Health” issue (6). In animal health, antibiotics are mainly used as “prophylactics” and/or for growth promotion. Indeed, antibiotic use in animals and agriculture accounts for ~70% of total antibiotic consumption (7–9). Pharmaceutical companies are investing less in the development of new antibiotics. Because of this, the discovery rate of new products has dramatically decreased since the 1980s and there have been few if any recent breakthroughs, reducing the options for treatment (10).

### A Multisectoral Global Action Plan to Contain Antimicrobial Resistance

In view of the growing problem of AMR, the World Health Organization (WHO), the Food and Agriculture Organization (FAO) and the World Organization for Animal Health (OIE) recently (2015) joined forces. Together, they developed the Global Action Plan on AMR (Box 1) (11, 12), which provides a framework for developing multisectoral national action plans (13).

Box 1 **The five strategic objectives of the “Global action plan for antimicrobial resistance” of the World Health Organization^2^**

The plan fits into the “One Health” approach and the tripartite of WHO, Food and Agriculture Organization (FAO) and the Organization of Animal Health (OIE) and was adopted by the May 2015 World Health Assembly (A68/20)^3^

1. Improve awareness and understanding of antimicrobial resistance through effective communication, education and training.
2. Strengthen the knowledge and evidence base through surveillance and research.
3. Reduce the incidence of infection through effective sanitation, hygiene and infection prevention measures.
4. Optimize the use of antimicrobial medicines in human and animal health.
5. Develop the economic case for sustainable investment that takes account of the needs of all countries, and increase investment in new medicines, diagnostic tools, vaccines and other interventions.

Clinical bacteriology at the point of referral hospitals in Low Resource Settings is pivotal for antimicrobial resistance surveillance (Objective 2) which contributes to awareness and understanding (Objective 1). Clinical Bacteriology Laboratories further are instrumental to infection prevention and control (IPC, preventing spread of multidrug resistant bacteria, Objective 3) and correct use of antibiotics (Antibiotic Stewardship, contributing appropriated antibiotic use, Objective 4). Of note, FAO and OIE have similar approaches for their sectors.

### Low-and Middle-Income Countries Are Hit Hardest by Antimicrobial Resistance

Although data are scarce and incomplete (particularly for Sub-Saharan Africa), it is assumed that LRS are hit hardest by AMR in terms of mortality, morbidity and associated costs (14–16). However, most information comes from hospital-based studies in high-risk patient wards (e.g., intensive care units), which complicates the evaluation of the impact of AMR. A systematic review of the available studies showed that AMR in “ESKAPE” bacteria is significantly associated with increased mortality. These “ESKAPE” bacteria include *Enterococcus* spp., *Staphylococcus aureus, Klebsiella pneumoniae, Acinetobacter baumannii, Pseudomonas aeruginosa*, and *Enterobacter* spp. These bacteria are listed by the WHO as “priority pathogens” for research on the development of new antibiotics and control measures (17).

AMR also occurs outside the hospital. On the one hand, high proportions of multidrug-resistant bacteria have been found in the commensal flora of humans (*Escherichia coli* and *Klebsiella pneumoniae*) and in the environment, including sources of drinking water (18, 19). On the other hand, typical community-associated pathogens, such as *Salmonella* Typhi (causing enteric fever), have acquired multidrug resistance, even against key antibiotics such as ceftriaxone (20, 21). Moreover, in November 2016, the health authorities in Pakistan reported an (ongoing) outbreak of typhoid fever caused by a strain of *Salmonella* Typhi resistant to all recommended antibiotics (termed “extensively drug resistant” or XDR). In December 2018, 5,274 cases had already been identified (22). It was of concern that the XDR Typhi strains from DR Congo and Pakistan acquired the genetic code for ceftriaxone resistance by horizontal transmission from common enteric bacteria (*E. coli* and *Klebsiella* spp.) distributed worldwide (20, 23).

Table 1 lists the determinants and aggravating factors that explain the vulnerability of LMIC to AMR. Beyond the well-known challenges of budgets and means, beliefs and social norms also guide the health-seeking itineraries of patients. These can partially impede diagnosis. A lack of diagnostic support leads to diagnostic uncertainty, which results in the over-treatment of patients with broad-spectrum antibiotics. Because of the poor implementation of infection prevention and control (IPC) measures, this promotes the emergence and spread of multidrug resistant (MDR) organisms.

**Table 1:** Why low-and middle-income countries are hit hardest by antimicrobial resistance: attitudes, practices, and interactions between prescriber, dispenser, patients, diagnostics and health systems, related to human medicine^4^ (4, 24–36).

**Prescriber considerations:** ∘ Absence of local surveillance data obscures awareness and knowledge about AMR in the own practice (“No data, no problem”) ∘ Poorly educated and trained in antibiotic use (see below) ∘ In the absence of diagnostic tools, he/she prefers to “cover” the patient for bacterial infections, preferably with broad-spectrum antibiotics ∘ Overuse but also suboptimal use: incorrect diagnosis, incorrect dose, timing, route, frequency and duration, no de-escalation (i.e., using an antibiotic of narrow spectrum based on microbiology reports) ∘ Extended use of antibiotics e.g., in the case of surgical prophylaxis (to “compensate” for inadequate infection control) ∘ The “Knowledge gap”: knowing that antibiotics are not indicated but nevertheless prescribing them (“cough and cold,” watery diarrhea) ∘ Fear of non-respecting and losing the patient when not prescribing antibiotics (taking his/her complaints not serious) ∘ Reliance on (own) clinical diagnosis	**Patient's attitudes, beliefs and socio-cultural factors:** ∘ Poor health literacy ∘ Out-of-pocket expenditure of healthcare costs ∘ Reluctance to blood sampling ∘ Patient or caretakers' pressure toward antibiotics (real or perceived by the prescriber) ∘ Auto-medication, non-prescription use of antibiotics (frequently associated with too low dose and too short duration) ∘ ABs are associated with power (strong, almost magical) and valued higher than the doctor's visit ∘ Poor awareness and knowledge about AMR: “the patient becomes resistant, not the bacteria,” ∘ “antibiotics protect against unsanitary conditions in the environment” ∘ Lay advice about antibiotics (friends, relatives) ∘ Storage of antibiotics left-overs at home—self/family medication ∘ Incorrect use—mixed with practices of traditional medicine
**Dispenser and supply** ∘ Poor access to antibiotics, inadequate supply leading to incorrect dose, timing, duration. ∘ Few professional pharmacists (pharmacy attendants, drug sellers) ∘ Economic incentives—e.g., selling particular brands ∘ Wants to fulfill the patients' demand: non-prescription sales of antibiotics, selling incomplete treatments, fear patient would go elsewhere ∘ Substandard (low content, expired, degraded) and falsified ABs	**Diagnostics at the first line:** ∘ Moderate to low clinical competence among frontline health workers ∘ POC testing for malaria in the absence of diagnostic algorithms for other febrile diseases has increased antibiotic prescription ∘ POC testing is not always accepted as part of a patients' consultation (financial reason or uncertainty of interpretation)**at the second and third line:** ∘ Few CBL, low volumes, low quality, not embedded in patient care
**Health systems:** ∘ Distrust in the quality of public (government-run) services ∘ Private market notably insensitive to regulation ∘ National Action Plans on AMR not yet developed or implemented ∘ Regulation (medicines, diagnostics) fragmented and poorly implemented ∘ No health insurance, “out of pocket” payment leads to underdiagnosis and under- or overtreatment	**Healthcare facilities:** ∘ Few or no programs of antibiotic stewardship available ∘ Poor infection prevention and control, fueling transmission of MDR organisms in hospitals, in turn increasing the use of antibiotics ∘ Payment per act in hospitals (consequences of AMR less visible)
**Education of providers and prescribers:** ∘ Professional education not adapted to needs) ∘ No registration, re-certification or continuing medical education ∘ Gaps in teaching of clinical microbiology and antibiotic prescription ∘ Poor awareness of local/national prescribing guidelines ∘ High influence of pharmaceutical drug promotion/representatives ∘ Hierarchic role model: respect of senior medical staff, reluctance to question prescribing decisions ∘ Autonomy of decision making (particularly in private hospitals)	**General:** ∘ Diagnostics market in low-and middle-income countries is uncertain ∘ Diagnostics for bacteriological cultures are considered as “low risk” products, hence low regulatory stringency ∘ No (supra)national “vertical” control programmes ∘ Fragmented donor landscape with competing interests

*AMR, antimicrobial resistance; CBL, clinical bacteriology laboratory; MDR, multidrug resistant; POC, point-of-care testing*.

## The Need for Bacteriology Laboratories at the Secondary Healthcare Level is Now Recognized

*Clinical bacteriology laboratory services are traditionally restricted to higher healthcare levels, where they are poorly linked to clinical services and therefore underused. Last year, the WHO recommended that clinical bacteriology services be available at the hospital level and that sampling be conducted at the primary level. This offers opportunities for expanding surveillance networks*.

### The Hierarchy of the Health Laboratory System at the Country Level

**Figure 1:**
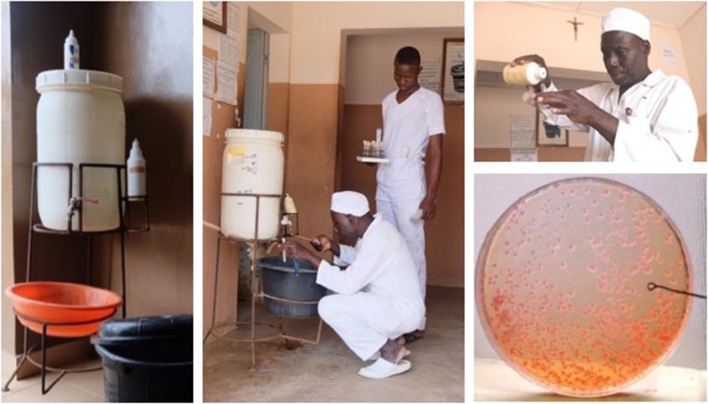
Benin, West-Africa: mobile hand washing facility. The water in the reservoir is a 0.05% chlorine solution which was added when the reservoir was nearly empty. Two containers with liquid soap (top and right side of the reservoir) were topped-up when needed. Simple swabbing of the tap and semi-quantitative culture of the soap (calibrated loop) was performed on standard culture media (MacConkey agar). Tap and soap were heavily contaminated with multidrug resistant *Klebsiella pneumoniae*. Simple control measures (daily cleaning and drying of the reservoir (system of alternating two reservoirs), replacing the containers instead of topping-up) stopped the contamination. Follow-up cultures were done during the implementation phase of the control measures. Written informed consent was obtained from the individual for the publication of this image.

Figure 1 depicts the levels of healthcare with a focus on the diagnosis and treatment of bacterial infections. Basic levels of healthcare (provided by community health workers, health posts, and health centres) are characterized by the limited clinical expertise of frontline healthcare workers. This results in a failure to assess alarming clinical symptoms and perform triage, which in turn leads to aggravated consequences for bacterial infections (24, 37). The secondary level of healthcare (representing the first referral level) accommodates medical laboratories with trained laboratory technicians. These laboratories refer to regional or provincial laboratories, which in turn refer to national reference laboratories (highest level).

### The Placement of Clinical Bacteriology Laboratories Where They Are Needed at the Level of the Referral Hospital

Until recently, guidelines for national and transnational laboratory networks advocated for CBL at the provincial and national referral levels (38–40). In Sub-Saharan Africa, these higher-level laboratories are traditionally poorly linked to clinical services and are therefore underused (41). In April 2018, the WHO Strategic Advisory Group on *in vitro* Diagnostics (SAGE-IVD) published the Model List of Essential *in vitro* Diagnostics (WHO EDL), which placed the culture-based diagnosis of invasive bacterial infections (such as bloodstream infections) at the second level of healthcare (i.e., at the referral hospitals) (42). This confirms the relevance of the few CBLs already implemented in LMIC hospitals (see below) and appeals for the systematic installation of CBL at the hospital level (43). Furthermore, the WHO EDL recommends the sampling of cultures at the primary level and their transport to the secondary level for processing and diagnosis (38, 42). This is already possible using current tools (44).

**Figure 2:**
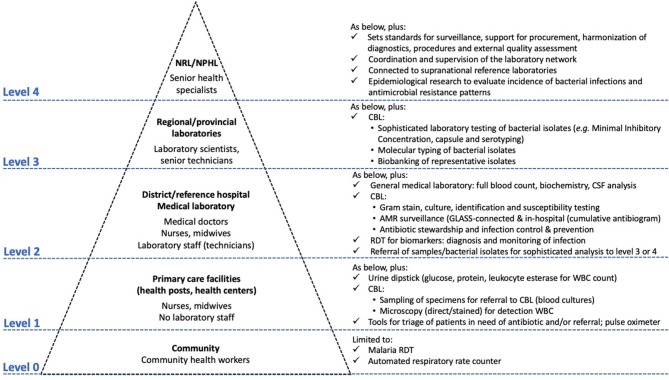
The integrated Tiered Laboratory Network with focus on test menus relevant to clinical bacteriology at different levels-of-care. Clinical bacteriology has recently moved from Level ≥ 3 to Level 2, i.e., the district or referral hospital. Adapted from Unicef and WHO (37), Best and Sakande (39), WHO (47), Centers for Disease Control and Prevention (48), Unitaid (24), and WHO (49), which provide complementary information for the tiered work-up of HIV, tuberculosis and malaria and other diagnostics. CBL, clinical bacteriology laboratory; GLASS, Global Antimicrobial Resistance Surveillance System; RDT, rapid diagnostic test.

Moreover, placing CBL at the level of referral hospitals conforms strategically within the scope of the International Health Regulations (45) and the Global Health Security Agenda^5^, as laboratory data at this healthcare level constitutes early alerts of emerging epidemics (25, 46). In addition, this offers opportunities for expanding national AMR surveillance and providing antibiotic stewardship and IPC at the hospital level, thereby achieving four of the objectives of the WHO AMR action plan (Figure 2).

## The Clinical Bacteriology Laboratory Allows for Antimicrobial Resistance Surveillance

*Hospitals are at the frontline of the emergence and spread of AMR but are also ideally positioned for training professionals and the roll-out of AMR surveillance via connection to the Global Antimicrobial Resistance Surveillance System*.

Although hospitals account for only 10–20% of antibiotic use in human health^4^, they are at the frontline of the emergence and spread of AMR. The impact of infections with MDR bacteria is most visible in hospitalized patients, given the numbers of susceptible patients (HIV/AIDS, trauma, neonates, and immunocompromised patients) combined with the use of invasive interventions and surgery. In hospitals in LRS, this is further aggravated by the overuse of broad-spectrum second-line antibiotics to compensate for weak IPC programmes^4^, (10). Notwithstanding, hospitals are places of pre- and in-service training for health providers and facilitate educational and behavioral interventions.

### AMR Surveillance: Compiling Routine Patient Data

The results of culture and antibiotic susceptibility testing (AST) generated by CBLs can be exploited for AMR beyond their use in daily care. Compiling day-to-day laboratory reports in a continuous way is the basis for AMR. This was already endorsed by the WHO in 2001 as part of the WHO Global Strategy for the Containment of Antimicrobial Resistance (50). In high- and middle-income countries, it has become an established practice; examples of this include the European Antimicrobial Resistance Surveillance Network (51), the Central Asian and Eastern European Surveillance of Antimicrobial Resistance^6^ and the Latin American Antimicrobial Resistance Surveillance Network (52). Surveillance activities have also been conducted in hospitals (secondary or tertiary level) in LRS where CBL have been installed as part of operational research and capacity building programmes (53–56) and have been frequently organized into networks^7^.

### Connecting to the Global Antimicrobial Resistance Surveillance System (GLASS)

The Global Antimicrobial Resistance Surveillance System (GLASS), which was developed by the WHO (57), is a platform for designing and organizing AMR surveillance at the local and national levels that facilitates global reporting. In its current version, GLASS is confined to human pathogens. GLASS surveillance combines laboratory data from routine patient care with basic demographic and clinical data. Box 2 lists the key actors in GLASS at the country level and their respective tasks.

Box 2 **Country-based surveillance of antimicrobial resistance (AMR) according to the Global Antimicrobial Resistance Surveillance System (GLASS) developed by the WHO (11, 57): roles and tasks of the core components, priority specimens and pathogens.**

**Table:** 

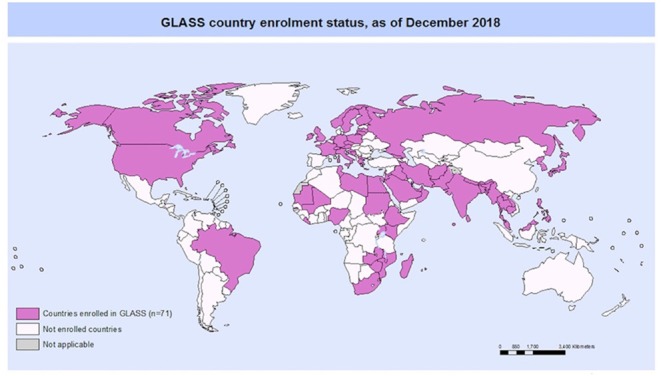 https://www.who.int/glass/country-participation/glass-map-1200px.jpg	Countries participating in GLASS commit to initiate or strengthen their national AMR surveillance system to generate quality AMR surveillance data to be shared internationally. In December 2018, 71 countries were enrolled in GLASS ∘ 39.4% high-income countries ∘ 25.4% lower-middle-income countries ∘ 19.7% upper-middle-income countries ∘ 15.5% low-income countries	
**GLASS core components:**	**GLASS priority pathogens:**	**GLASS priority specimens:**
**National Coordination Centre (NCC):**	°*Escherichia coli*	∘ Blood
∘ defines national AMR surveillance objectives	°*Klebsiella pneumoniae*	∘ Urine
∘ collects and aggregates data at the national level (including quality control and deduplication)	°*Acinetobacter baumannii*	∘ Stool
∘ reports every 12 months and as per national surveillance schedule	°*Staphylococcus aureus*	∘ Urethral swabs
∘ shares nationally aggregated data with WHO ∘ monitors and evaluates the national surveillance system	°*Salmonella* spp.	∘ Cervical swabs
**National Reference Laboratory (NRL)**	°*Shigella* spp.	
∘ provides guidance and technical support to surveillance sites: dissemination of standards, reference materials, procedures	°*Neisseria gonorrhoeae*	
∘ coordinates quality assessment in the AMR surveillance sites (organizes proficiency testing (external quality assessments)		
∘ provides confirmatory and extended microbiological testing		
**AMR surveillance sites:**		
∘ collects microbiological information		
∘ collects basic demographic, clinical and epidemiological information		
∘ verify, analyze and consolidate the surveillance data ∘ promote diagnostic stewardship activities to support patient care and surveillance		

The GLASS national coordination centre is usually a public health institute that defines, organizes and monitors national AMR surveillance. Whenever possible, it should be linked to sectors involved in animal and human welfare and agriculture. The national reference laboratory provides capacity building for the AMR surveillance sites and confirmatory testing. The AMR surveillance sites should constitute a network that represents the different geographic, demographic and socio-economic levels within the country and should provide access to epidemiological and laboratory support. Both in- and outpatient health facilities are eligible (11, 57).

Priority specimens comprise blood, urine, feces and urethral swabs, and the eight priority pathogens largely overlap with those in the “Priority Pathogen List for Research and Development of New Antibiotics” provided by the WHO (58). Antibiotics under surveillance include those listed as treatment options for priority pathogens or diseases in the WHO Essential Medicines List (59, 60) and potential reserve antibiotics and antibiotics used as indicator (surrogate) products (i.e., to predict susceptibility or resistance to other antibiotics).

For data management and transfer, national systems can be used, and the open-access WHONET software has been adapted to facilitate data entry at surveillance sites and (automated) processing at the national coordination centre^8^. In December 2018, a total of 71 countries participated in GLASS, of which 15 were in Sub-Saharan Africa^9^. GLASS is a response to Objective 2 of the WHO ‘Global Action Plan for Antimicrobial Resistance’ (surveillance of AMR and antibiotic consumption in all sectors), and GLASS data boosts awareness among providers, patients and the public (Objective 1).

### Additional Benefits Associated With Surveillance

Microbiological surveillance based on hospital data provides valuable information at different levels (10, 61). First, it provides opportunities to collect, assess, monitor and improve quality indicators, especially for blood cultures (62). Second, apart from AMR, it sets a baseline and provides monitoring of the spectrum of bacteria causing infections. Indeed, this spectrum can vary considerably in different sites and settings. Surveillance can therefore determine the occurrence and proportions of geographically confined pathogens such as *Burkholderia pseudomallei* (melioidosis, South-East Asia) (53), *Streptococcus suis* (zoonotic meningitis and sepsis, Asia) (55) and *Salmonella* species (both typhoidal and non-typhoidal) (54, 63, 64). Consistent blood culture surveillance as part of capacity building projects also allows for early alerts for hospital and community outbreaks (Box 3).

Box 3 **How Clinical Bacteriology can contribute to Diagnostic Stewardship in LRS hospitals**

According to the GLASS Manual (65), Diagnostic Stewardship is defined as: “*coordinated guidance and interventions to improve appropriate use of microbiological diagnostics to guide therapeutic decisions.”* The clinical bacteriology laboratory should work out guidelines covering the diagnostic pathway along request, sampling, laboratory processing and reporting. In addition to written guidelines, laboratory staff should be available on a consultative basis to discuss tailored adaptations (66). The text below comprises, in addition to Table 2, some practical guidance. There are no guidelines nor studies [except for reference (67)] specific for low resource settings, all references address high income countries.

**Table:** 

**1. Specimen selection and collection guidelines can be specimen-oriented (“blood, bone marrow …)** (68), or system-based (“bloodstream infections”), such as the Infectious Diseases Society of America (IDSA) 2018 guidelines (69) which provide interesting background clinical insights as well as guidelines for sampling in children. Guidelines should also detail instructions for labeling and request form and list specimen rejection criteria. **2. Laboratory working-up should be evidence-based and cost-efficient.** - Abbreviated work-up and identification is recommended (27, 70) - Prioritize specimens according to clinical impact and feasibility (27) - Urine: minimal work-up of low organisms counts, no work-up when ≥ 3 species present - Urine cultures can be limited by pre-testing for white blood cells (dipstick) (71) - Wound, mixed flora: work-up only predominant organism guided by Gram stain - Anaerobic cultures are demanding, in case of mixed flora of limited added value - Respiratory tract specimens: do not process if excess of epithelial cells (contamination) **3. Apart from appropriateness and accuracy, timeliness is key** Considerable shorting of turnaround time can be achieved in the chain from collection to reporting (e.g., “needle to incubator time”) (72–74) **4. Common examples of “do's and don'ts” in specimen management” (4, 69):** Do not submit (because clinically irrelevant and potential misleading): - Urine or drain fluid from catheter bag (bacteria grown in the bag) - Urine or drain catheter tip (colonizing flora) - Blood cultures sampled through indwelling venous catheter (skin flora) - Endotracheal tubes (colonizing bacteria) - Superficial wound cultures (colonizing bacteria) - “Wound cultures” in case of uncomplicated drainage of skin - or soft tissue abscess Do not work-up “everything that grows”(69): Do not work-up colonizing flora: respiratory tract, fistulae, superficial wound swabs … Do not work-up contaminating flora: e.g., skin contaminants in blood cultures A swab is not a specimen of choice (69) - Submit pus, aspirate or tissue, not a swab - Non-flocked swabs contain too small volumes with irregular release on agar plates Obtain specimen before administration of antibiotics **5. Work-out a “send-out catalog” with the National Reference Laboratory (NRL):** Suspected isolates of biosafety or epidemic impact (many are “tropical”), e.g., *- Salmonella* Typhi, *Brucella* spp., *Bacillus anthracis, Vibrio cholerae* … (75) Sophisticated testing and typing, e.g., - Minimal inhibitory concentration of ciprofloxacin for *Salmonella* Typhi - Bacteria from healthcare-associated outbreaks **6. Diagnostic stewardship guidelines should be drafted with participation of clinicians.** Failing to do so may avoid frustrations and non-compliance from clinicians because of perceived reduction in clinical autonomy as well as unpleasant discussions (70, 76). Examples are: - Definition and limited work-up of contaminants - Storage of original samples, Gram stain slides, initial cultures and strains for look-back or extended testing **7. Guidelines by themselves are not sufficient, and training of all cadres (clinician, nursing staff and laboratory staff is needed).** The nursing staff is “caught in the middle” between clinicians and laboratory but has a pivotal role in selection, collection and transport of samples (69). In high income countries, requesting irrational (unnecessary) testing may be due to fear to miss diagnosis, defensive medicine, fear of litigation and academic curiosity (70). In low resource settings, over-prescribing may be a problem too (67). Clinicians-in-training may be more than practicing clinicians inclined to adapt prescribing behavior (70). **8. Training should be supported by administrative controls and monitoring**, e.g., Defining and describing roles and responsibilities of clinical, laboratory and nursing staff. Monitoring of quality indicators in the scope of a quality improvement project such as - blood cultures: proportion of skin contaminants (62) - quality of labeling and request forms (67) - turn-around time of alert reports - non-collected report forms (67) **9. Diagnostic Stewardship is most effective as part of a comprehensive antibiotic stewardship program (76)**.

## The Clinical Bacteriology Laboratory Contributes to Antibiotic Stewardship

*Antibiotic stewardship (ABS) aims to optimize the treatment of infections and reduce adverse events due to antibiotics. In addition, ABS programmes are effective in reducing AMR at the hospital level*. *Table 2* *gives an overview of the core elements of ABS along with contributions by CBL, most of which have been studied and applied in high-income countries. In this section, we outline processes to be considered when implementing CBL to maximize the impact on ABS*.

**Table 2:** How the clinical bacteriology laboratory can contribute to antibiotic stewardship in hospitals.

**Core element**	**Actions to be taken**
**Elements 1, 2 and 3: Leadership commitment, Accountability, Drug Expertise** • Formal, written statement of support from leadership for ABS • Budgeted financial support for ABS activities • Physician leader responsible for program outcomes of ABS • Pharmacist leader involved in ABS	**Key support of hospital staff collaborating with the ABS leaders** ∘ Clinicians and department heads ∘ Infection Prevention and Healthcare Epidemiology ∘ Quality Improvement ∘ Nursing staff ∘ Link doctors and link nurses in every hospital ward ∘ Laboratory^*^: **Diagnostic stewardship**: proper use of diagnostic tests and flow of results: - Guidance for sampling (indications, specimen, timing, precautions, transport - Establish and implement sample rejection criteria - Timely transport, organize reception, routing and tracking of the samples in the laboratory - Assure accurate state-of-the-art processing of submitted samples - Prioritizing samples for quick processing and reporting - Performing rapid diagnostic testing (biomarkers, molecular and antigen-based diagnosis) - Timely reporting of actionable intermediate and preliminary results - Enhanced and clinically relevant final report - “Liaison” with clinicians and nursing staff in high risk wards (intensive care unit) - Supplementary AST testing for newly introduced antibiotics - Referral of samples/isolates to reference laboratories for advanced level testing ∘ Information Technology (IT)^*^ - Reporting of preliminary results: text messaging, electronic messages - Reporting of final results: cascade/selective reporting, treatment-related comments - Digital clinical decision support systems at the bed-side or point-of-care - Transmission of AST data useful for pre-authorization of antibiotics to pharmacy - Transmission of AST data useful to guide review of antibiotics treatment - Creating prompts “pop-ups” e.g., “bug-drug mismatch” - Surveillance data, cumulative antibiogram report, antibiogram - Broad interventions and Pharmacy-driven interventions
**Element 4: ActionsPolicies** ∘ Prescribers document dose, duration, and indication for antibiotics prescriptions ∘ Hospital specific antibiotics treatment recommendations for common clinical conditions^*^	**Interventions** ***4. 1. Broad interventions*** ∘ Review of appropriateness of all antibiotics 48–72 h after the initial orders^*^ - antibiotic time out (ATO); “start smart, next focus,” Day 3 Bundle - degressive, cascade or selective reporting ∘ Specified antibiotics need to be approved prior to dispensing (pre-authorization)^*^ ∘ Review of antibiotics treatment for specified antibiotic agents^*^ - prospective audit with feedback, - alerts for duplicate antibiotic coverage - ward visits with face-to-face discussions by a dedicated team (“A-Team”) (Dick2015) ***4.2. Pharmacy-driven interventions*** ∘ Automatic changes from intravenous to oral antibiotic therapy^*^ ∘ Dose adjustments in cases of organ dysfunction ∘ Dose optimization (pharmacokinetics/pharmacodynamics) ∘ Automatic alerts in situations where therapy might be unnecessarily duplicative?^*^ ∘ Time-sensitive automatic stop orders for specified antibiotic prescriptions? ***4.3. Specific diagnosis and infections:*** **interventions to ensure optimal antibiotics use for treatment of:** ∘ Community-acquired pneumonia^*^ ∘ Urinary tract infection^*^ ∘ Skin and soft tissue infections^*^ ∘ Surgical prophylaxis ∘ Empiric treatment of Methicillin-resistant *Staphylococcus aureus* (MRSA) ∘ Non-*Clostridium* difficile infection (CDI) antibiotics in new cases of CDI ∘ Culture-proven invasive (e.g., blood stream) infections^*^
**Element 5: Tracking: monitoring antibiotic prescribing, use and AMR resistance process measures**	***5.1. Process measures*****:** ∘ Monitoring adherence to a documentation policy (dose, duration, and indication) ∘ Monitoring adherence to hospital-based antibiotic treatment guidelines ∘ Monitoring compliance with interventions in place ***5.2. Outcome measures*** ∘ Tracking rates of *Clostridium difficile* ∘ Producing and diffusing surveillance data (e.g., cumulative antibiogram) ***5.3. Antibiotic consumption measures*** ∘ Monitoring counts of antibiotics administered to patients per day (Days of Therapy; DOT) ∘ Monitoring grams of antibiotics used (Defined Daily Dose, DDD) ∘ Monitoring direct expenditure for antibiotics (purchasing costs)
**Element 6: Reporting information to staff**	∘ Share facility-specific reports on antibiotics use with prescribers ∘ Distributing Cumulative Antibiogram results to prescribers and management at the hospital^*^ ∘ Direct, personalized communication with prescribers about antibiotic prescribing^*^
**Element 7: Education**	∘ Providing education to clinicians and other relevant staff on antibiotic prescribing^*^

Actions to be taken are listed according to the U.S. Centers for Disease Control “Core elements of Hospital Antibiotic Stewardship Programs” (77). According to British society for antimicrobial Chemotherapy (4), Dyar et al. (78), Patel and Fang (79), Morency-Potvin et al. (80), CDC (77, 81), Bouza et al. (82), Dellit et al. (83), Macvane et al. (84), Trivedi and Kuper (85), Cosgrove et al. (86), Versporten et al. (87), Donnell and Guarascio (88), Pulcini and Gyssens (89), Pardo et al. (90), and Baron et al. (91).

* Particular tasks and roles for the Clinical Bacteriology Laboratory in Low Resource Settings.

*ABS, antibiotic stewardship; AST, antibiotic susceptibility testing; CDI, Clostridium difficile; CBL, clinical bacteriology laboratory*.

### Diagnostic Stewardship

Diagnostic stewardship involves guidelines about the appropriateness of diagnostic tools, specimen selection, the timing and conditions of sampling, the understanding of sample flow and processing, and the correct interpretation and reporting of results. This ultimately leads to the optimization of patient diagnosis, antibiotic treatment and outcomes, resulting in a decrease in AMR (66, 76, 78, 79). To implement diagnostic stewardship, the guidelines should be available in writing, and competent laboratory staff must be available to address questions and comments. Box 4 gives an overview of the basic guidelines of diagnostic stewardship for CBL.

Box 4 **How clinical bacteriology can benefit from the achievements in laboratory strengthening of vertical disease programs (HIV, tuberculosis, and malaria) in Sub-Saharan Africa.**

The integrated laboratory strengthening approach in Sub-Saharan Africa was initiated by advocacy expressed in the Maputo Declaration in 2008 and mobilized major funders such as the US President's Emergency Plan for AIDS Relief (PEPFAR), the Global Fund for AIDS, Tuberculosis and Malaria and the World Bank toward an unprecedented support for vertical health programs. It uses innovative approaches for teaching and accreditation (SLIPTA and SLMTA) and is coordinated by WHO Regional Office for Africa (WHO AFRO) and the African Society of Laboratory Medicine (ASLM) at the regional level and by national laboratory plans at the countries' level. The laboratory strengthening for the vertical disease programs of HIV, tuberculosis and malaria is organized along country's National Public Health Laboratory and tiered integrated laboratory networks. It aims to support to other disease surveillance systems (Freetown Declaration 2015) within the Global Health Security Agenda.

Below are listed 10 key-factors to success on which laboratory medicine in general and clinical bacteriology in can capitalize for implementation. Adapted from Nkengasong et al. (41), Barbé et al. (92); Alemnji et al. (93), Nkengasong et al. (94), Andiric et al. (95), WHO (96); Carter et al. (97), and Sayed et al. (98).

1. Advocacy
  – The advocacy role from Africa-owned platforms is essential to raise funding and setting the agenda
  – Examples; Maputo Declaration, Freetown Declaration
2. Commitment of funding by donors
3. Involve the private sector
  – The premium for-profit private sector may have better access to advanced technologies and staff
  – Non-governmental organizations and mission hospitals reach rural and under-served communities
  – Postal and courier services can be contracted for safe and efficient shipment of samples
4. Effective partnerships coordinated at the national level: Embedded in the National Laboratory Strategic Plans
5. Tiered and Integrated referral networks overseen by National Reference Laboratories
6. Promote the general quality management system of the laboratory and accreditation: WHO-AFRO Stepwise Laboratory Quality Improvement Toward Accreditation (SLIPTA) program
7. Enhance education and training by innovative tools: WHO-AFRO Strengthening Laboratory Management Toward Accreditation (SLMTA) toolkit
8. Investment in infrastructure of laboratories: Infrastructure covers facilities and safety, equipment, consumables, process and information management, procurement, supply, and inventory systems
9. Care for the laboratory workforce: In-service training, retention strategy, career paths
10. Metrics allow for monitoring: Clear metrics for assessments and monitoring allow to measure improvements.

WHO-AFRO: World Health Organization Regional Office for Africa.

https://www.who.int/diagnostics_laboratory/Maputo-Declaration_2008.pdf

http://www.aslm.org/what-we-do/global-health-security/freetown-declaration/

http://www.aslm.org/what-we-do/slipta/

### Timely Transport and Accurate Processing of Submitted Samples and the Prioritization of Workflows

CBL addresses multiple parameters and procedures. Consequently, laboratory work-up processing is more prone to inter-operator variability and errors compared to other procedures of the medical laboratory (92, 99). Moreover, automation and batch sample processing are not feasible in most LRS settings. An integrated laboratory quality management system combined with bench-side supervision is therefore key to a well-functioning CBL (92). A competent CBL is also in close communication with care providers, including clinicians and nursing staff. Nurses are in charge of appropriate and timely sampling as part of their daily duties, and they closely review patient medications and responses to treatment. In addition, they are well-placed to initiate discussions about antibiotics, including the “antibiotic time-out” and the switch from intravenous to oral treatment, and are key to informing and educating patients (77, 80, 81, 100, 101).

### Rapid Diagnostic Testing

Sophisticated molecular and immunochromatographic testing shorten the turnaround time for identification and, to a certain extent, AST, thereby allowing the timely adaptation of empirical antibiotic treatment (79, 82). These exciting new innovations boost the role of the CBL in ABS but are currently not feasible or affordable in LRS. Judicious use of abbreviated biochemical identification can shorten the time-to-identification (102). Likewise, the use of rapid diagnostic tests (*Salmonella, Burkholderia pseudomallei*) for grown blood cultures can substantially simplify lab processes (26, 27, 103), but their optimal integration within the CBL workflow requires further study. The European Committee on Antimicrobial Susceptibility Testing recently validated direct AST testing from grown blood cultures for key pathogens (104)^11^ even though it is difficult to achieve this considering office hours, it is an important step to shorten the turnaround time of AST testing.

### Communication and Reporting

Reporting should be timely, clinically relevant, understandable and actionable, and should be received at a time when treatment decisions can be made and executed. As an example, the reporting of a Gram stain result for a blood culture at the time of growth guides the well-informed clinician toward the identity and anticipated AMR profile of the causative bacteria (105). Moreover, the “enhanced” reporting of the identity and AST goes beyond simply stating “susceptible” or “resistant.” It involves guidance for the interpretation of results, such as the prediction of cross-resistance. Conversely, not reporting AST results for colonizing or contaminating flora is helpful in decreasing antibiotic overuse (106). Examples of treatment-related comments are the so-called “expert rules,” which use the AMR patterns of bacteria to predict and report their cross- or co-resistance to other antibiotics or to alert for possible identification errors. Standard reporting formulas for interpretation and treatment comments are available (80, 106, 107); however, they need to be verified for comprehensibility and acceptability on site.

### Connectivity and Quality Assurance: The Laboratory Information System

A laboratory information system (LIS) is a powerful tool for patient and sample management; in high-income countries it has been demonstrated to shorten time-to-result and timeliness of clinical responses as well as to improve laboratory quality (108–110). A strong and integrated LIS is further a crucial tool for supply and workload management, quality control and financial planning (111). For instance, it can prevent errors in patient identification and transcription (92). LIS contributes to harmonized interpretation and consistent reporting. Examples are the reporting of preliminary results (e.g., electronic messaging and text messaging), the application of expert rules and the addition of treatment-related comments (80). When connected to the patient's electronic medical record, the LIS can be integrated into digital decision support systems. In addition, a well-functioning LIS is pivotal for drug-based or pharmacy-driven ABS interventions such as alerts in cases of incorrect or duplicated antibiotic prescriptions (79, 80) and for aggregating laboratory data into a surveillance report or so-called cumulative antibiogram (see below). There are budgetary barriers for using LIS in LRS, since the hardware and network costs cannot be neglected. A substantial barrier is the cost associated with the introduction of the infrastructure and software (98). Free-of-charge LIS are available and have been listed earlier (92). However, the currently freely available LIS require technological knowledge, have poor standardization and have frequent breakdowns while offering insufficient support for maintenance (112). The creation of an open-source LIS, appropriate for LRS, is thus desirable.

### Specific Antibiotic Stewardship Interventions

A review of the appropriateness of empirical antibiotic treatment 48 h after initiation is an accessible and strongly promoted action for ABS. For this “antibiotic time-out,” timely information about CBL results is critical (77, 81). Typically, the “4 Ds” of ABS are reviewed: the diagnosis, the dose, the planned duration of antibiotic treatment, and the possibility of de-escalation (i.e., a switch to a narrow-spectrum antibiotic or to oral antibiotic treatment). Likewise, the so-called cascade reporting (degressive or selective) of AST is recommended. This means that the CBL reports AST results for second-line antibiotics only for bacteria that are resistant to first-line antibiotics. Cascade reporting provides a tool for streamlining antibiotic treatment from broad-spectrum to narrow-spectrum, thereby reducing antibiotic exposure and associated adverse events (83). Contextual barriers to AMS, such as physician awareness of antimicrobial options, the fear of retribution for adverse outcomes, and the degree of engagement of administrators and allied professions must be identified and addressed (113).

### Cumulative Antibiogram (Cumulative Antibiotic Susceptibility Report and Antibiogram)

The cumulative antibiogram is the product of AMR surveillance at the hospital level, as it combines culture and AST results for routinely submitted samples. It can be used to evaluate AMR for selected target bacteria for shorter periods and to stratify data at the ward level (114). The Clinical Laboratory Standards Institute provides extensive guidelines for developing and presenting cumulative antibiogram reports (115). However, processing raw laboratory data into a correct and readable report requires expertise (deduplication, verification, and expert rules) and is challenging (116, 117). The cumulative antibiogram offers a local “baseline AMR” allowing the hospital ABS team to adapt international and national antibiotic treatment guidelines to the local context. As an example, in a rural pediatric hospital located in the Democratic Republic of the Congo, cumulative antibiogram data led to improved antibiotic treatment of children admitted with bloodstream infections (118).

### Reporting of Information to Staff

The CBL should ensure the distribution of guidelines and reports (in particular the cumulative antibiogram) to care providers (clinicians and nursing staff), the hospital management and the ABS and IPC teams (see below) (119). Further distribution within the health hierarchy is valuable, as cumulative antibiogram data from samples from community-acquired infections can feed clinical algorithms and can be incorporated into electronic clinical decision support tools at the first level of healthcare (24).

### Education

Provider education is key to understanding and interpreting policies and actions related to ABS (120) and IPC (see below). There are many opportunities, formal and informal, for education in CBL. Beyond refreshing basic knowledge on bacteria and antibiotics in a contextual setting, efforts should also target the correct interpretation of cultures, AST results and cumulative antibiograms, as the in-depth understanding of trainees may be overestimated (120). Information may be diffused through posters and flyers and electronic newsletters, and CBL staff can contribute to the review of de-identified cases (121). Note that education by itself, although conducive to understanding, is not effective by its own and needs to be supported by more persuasive administrative tools (83).

## The Clinical Bacteriology Laboratory Contributes to Infection Prevention and Control

*Infection prevention and control programmes are effective tools to reduce health care-associated infections. The clinical bacteriology laboratory can contribute actively and passively to IPC activities. Key activities will be described in this section*.

Table 3 lists the tasks and roles of CBL in hospital IPC according to the WHO “Guidelines on the Core Components of IPC Programmes.” The Core Components are based on a systematic literature review and the Grading of Recommendations Assessment Development and Evaluation (GRADE) approach (119). Compared to the 2009 version, the 2016 WHO IPC guidelines have embraced AMR and focused on the integration of IPC with water, sanitation and hygiene (WASH), to which CBL can effectively contribute (119). Furthermore, depending on their training and competence, CBL staff can contribute to IPC guidelines (design, implementation and monitoring) and IPC educational activities (Core Components 2 and 3, respectively) (122). Education about the habitats and epidemiology of bacteria that cause health care-associated infections (HAI) is important for the understanding of IPC measures (123). In high-income countries, the CBL also contributes because clinical microbiologists trained in IPC often chair the hospital infection prevention and control committee (122, 124). In remote and small hospitals in LRS, skilled microbiologists, however, are rare (see below).

**Table 3:** How the Clinical Bacteriology Laboratory can contribute to Infection Prevention and Control in hospitals.

**Core element**	**Actions to be taken**
1. IPC programs	IPC program with a dedicated trained team including microbiology^*^
2. IPC guidelines	Evidence-based guidelines based on international standards and adapted to local conditions
3. IPC education and training	Concepts and theories of microbiology, infectious diseases and IPC^*^ Diagnostic stewardship (see Table 2) Assisting in interpretation of cultures (e.g., colonizing vs. infecting bacteria)
4. Health care-associated infection surveillance	Active surveillance of HAI: IPC team driven, focusing on ward, site or priority^*^ Communication to the IPC team of bacteria of IPC interest, e.g., for contact, droplet, and airborne isolation precautions^*^ (in some settings: conducting active screening cultures for colonization with MDR bacteria) Passive surveillance of HAI: laboratory driven, based on routinely submitted samples^*^ (see Table 2, Cumulative antibiogram) Early warning for hospital-acquired outbreaks, suspicion in case of^*^: - ESKAPE bacteria: *Enterococcus* spp., *Staphylococcus aureus, Klebsiella pneumoniae, Acinetobacter baumannii, Pseudomonas aeruginosa, Enterobacter* spp. or non-fermentative Gramnegative rods (*Burkholderia cepacia, Stenotrophomonas maltophilia …*), frequently MDR - recovered from normally sterile body fluids (blood, urine …) - mostly from patients in high-risk areas (intensive care, neonatology, invasive procedures) - frequently in clusters (common-source) or series (propagated transmission) of patients Assist in the investigation of HAI and outbreak management (reservoir and transmission)^*^ - Conduct (directed) environmental sampling and processing - Submit bacteria from patients and environment to reference laboratory for typing Processing and monitoring biological indicators of sterilization Monitoring the microbiological quality of^*^: - Consumption water, particularly in high risk wards (operating room, nebulization, oxygen concentrator, dialysis)^*^ - In-house prepared or distributed disinfectants and antiseptics^*^ - In-house prepared or distributed food (neonatology, malnutrition kitchen)
5. Implementation of IPC: multimodal strategies	Outcome and changing-behavior approach bases on (i) system change (availability of infrastructure and supplies, (ii) education and training, (iii) monitoring, (iv) reminders at the workplace and (v) culture change/safety climate in the hospital.
6. Monitoring, evaluation and feedback^*^	Achieve behavior changes and process modifications through continuous monitoring
7. Workload, staffing and bed occupancy	Ward design, bed occupancy, staff indicators
8. Built environment, materials and equipment for IPC including hand hygiene	Clean and hygienic hospital environment conducive to IPC practice Assuring safe and effective WASH (water, sanitation, hygiene) (see 4)^*^

*Actions to be taken are listed according to the World Health Organization's “Guidelines on Core Components of Infection Prevention and Control Programs at the National and Acute Health Care Facility Level” (119). According to WHO (119), Stevens and Wenzel (122), Tacconelli et al. (123), Friedman (124), CDC (125), and Miller (126)*.

* Particular tasks and roles for the Clinical Bacteriology Laboratory.

*HAI, health care-associated infections; IPC, Infection Prevention and Control; MDR, multidrug resistant (resistant against multiple antibiotic classes (127), WASH, water, sanitation and hygiene*.

IPC surveillance is an effective tool to monitor and reduce HAI (119). CBL can contribute to active, IPC team-initiated surveillance activities, which may be ward- or procedure-specific (e.g., focused on the intensive care unit or on catheter-related bloodstream infections, respectively) (119). Although CBL can improve the standardization and accuracy of IPC surveillance definitions (119), clinical specimens for CBL should be carefully selected in light of diagnostic stewardship. As an example, cultures grown from superficial wound swabs and urinary samples obtained from indwelling catheters are not per se indications of a healthcare-associated infection, given the (abundant) presence of colonizing and contaminating bacteria (Table 2) (4). In addition, the CBL needs to alert the IPC team in time of patients infected or colonized with bacteria subject to isolation precaution practices (“alert bacteria”), such as contact isolation for MDR bacteria (122–124).

Passive surveillance is CBL-initiated and consists of collecting routine cultures and AST data periodically. This can provide ward-stratified information about HAI and their AMR profiles, which in turn helps to define priorities for IPC programmes and allows the benchmarking and monitoring of IPC activities (119). In addition, the daily review of CBL data may offer early warnings of outbreaks of HAI (119); the epidemiological thresholds of healthcare-associated outbreaks may be low [>1 patient in case of group A post-puerperal infection (128)], but microbiological and basic clinical clues (invasive devices and procedures) may raise suspicion (Table 3). As is the case for ABS, this is part of the “liaison” between CBL and IPC teams, which is characterized by bidirectional communication and good interpersonal relations (124, 126).

The key activities of CBL for IPC include obtaining and processing samples to investigate the reservoirs and transmission routes of healthcare-associated outbreaks (119)^12^. The selection of sampling sites and items in the hospital environment should be directed by a competent member of the IPC team. Epidemiological data and observations of the patient-care setting can guide the sampling (122)^12^, which should take place as soon as possible during the outbreak. Whenever possible, molecular typing of outbreak-related bacteria should be performed to identify its relatedness with clinical bacteria (for instance, in the national reference laboratory). In the absence of such “molecular fingerprinting,” phenotypical aspects (rare species and specific biochemical and AST patterns) may be indicative of the role and identity of the causative organism (124)^12^. Screening for carriers (in stool and nasal swabs and on finger tips) among patients and providers should be restricted (122), particularly in the context of LRS. Likewise, “routine” environmental surveillance (i.e., sampling beyond the context of outbreak management, research, or educational purposes) is not recommended (122, 129).

Regarding WASH, the CDC guidelines recommend monitoring water used for dialysis (122)^12^. Given the serious problem of safe water in health facilities in LMIC^13^, CBL can also conduct monitoring of the bacteriological quality of water and water-derived products, particularly for high-risk patients and procedures. Notable sources of healthcare-associated outbreaks in LRS include inappropriately used “multidose” or “multiple-use” vials of reconstituted medicines and contaminated in-use disinfectants and soaps (130, 131). They are frequently overlooked as serious risks, as are other practices embedded in nursing care in LMIC (132). Bacteriological cultures obtained from such contaminated materials often constitute an “eye opener” for staff and can contribute to better risk perception (Figure 1).

## What Can the Clinical Bacteriology Laboratory Learn From the HIV, Tuberculosis and Malaria Programmes?

*Due to initiatives in the treatment of HIV, tuberculosis and malaria, improvements in laboratory medicine in Sub-Saharan Africa have been observed in recent years. The ways in which clinical bacteriology have benefited from these achievements is described in the following section*.

### Laboratory Medicine in Sub-Saharan Africa: Progress in HIV, Tuberculosis, and Malaria

Laboratory services for the diagnosis and control of HIV, tuberculosis and malaria (commonly known as “the big three”) in Sub-Saharan Africa have been transformed and tremendously improved (14). Through the improvement of national reference laboratories, a network of diagnostic services has been built and coached toward accreditation (93, 94). However, this “*decade of remarkable progress*” (93) has mainly been confined to the aforementioned vertical disease programmes. Moreover, a recent series of personal review papers has deemed pathology and laboratory medicine (“PALM”) services in Sub-Saharan Africa as insufficient in terms of scope, access, and quality (25). Clinical bacteriology was evaluated as the most lacking in quality management and as showing inadequate performance (92, 133). Table 4 lists the challenges and requirements for the sustainable implementation of medical laboratories at the programmatic (PALM) and operational (CBL) levels.

**Table 4:** Challenges and needs for implantation of Clinical Bacteriology Laboratories at the hospital level in low resource settings, specifically in Sub-Saharan Africa.

**Requirements for implementing “PALM” (25, 28, 98) =pathology and laboratory medicine**	**Requirements for implementing clinical bacteriology (27)**
Human Resources Education and training Infrastructure: Facilities, equipment, consumables, biosafety, supply, process, and information management Quality, standards, accreditation	Tropicalization of diagnostics Embedment in clinical care Adapted training materials Rationalized Identification and antibiotic susceptibility testing Selection of specimens On-site validation and field adoption of new diagnostic tests

*The left column lists the requirements at the general medical laboratory including pathology, the right column focuses on clinical bacteriology*.

### What Can Clinical Bacteriology Adopt and Learn From This Progress?

The above-described progress in the vertical “big three” laboratory services was based on advocacy, global investment, laboratory innovation, and common commitment (41). Although the intended spill-over of laboratory capacity improvements to horizontal health services has been modest (92), clinical bacteriology can benefit from these achievements. Box 4 lists examples based on recent references (41, 92–95, 98).

### Advocacy by Leading African Organizations

Advocacy is fundamental. The African Society for Laboratory Medicine (ASLM)^14^ is a pan-African organization for laboratory professionals endorsed by the African Union, which “coordinates, galvanizes and mobilizes relevant stakeholders to improve local access to world-class diagnostic services.” Launched in 2011, ASLM has been essential in strengthening the organization of competent laboratory services embedded in national networks and coaching them toward accreditation. Although initially focused on the “big three,” ASLM currently addresses the entire spectrum of laboratory medicine and has embraced AMR surveillance, ABS and IPC (134–136).

### Donor Commitment and Coordination

Likewise, donor commitment is important. Thematic donor support for containment is starting up (28), but as AMR crosses disciplines and sectors, donors may be more fragmented and dispersed compared to the “big three” main funding agencies (24). Importantly, donor support and partnerships need to be coordinated at the national level, and country leadership is crucial. Furthermore, the private sector needs to be engaged from the beginning, as private laboratories play a notable role as providers of laboratory medicine in Sub-Saharan Africa (98, 137). In close association are research groups conducting operational research in surveillance, ABS and IPC (96). HIV programmes have benefited from public-private partnerships to provide laboratory diagnosis and monitoring, but it is recommended to engage in clear communication and to explicitly define the role of each partner (94). A similar scenario can be considered for CBL, taking into account some particularities. For instance, given the wide range of consumables combined with the need for rationalized testing (27), equipment leasing using reagent-based contracts on an exclusive supplier basis may reduce the flexibility and autonomy of CBL and drive up costs.

### Tiered and Integrated Laboratory Networks

The strengthening the laboratory services in Sub-Saharan Africa since 2008 has utilized tiered and integrated laboratory networks that are overseen by National Reference Laboratories, which are in turn interconnected at the supranational (e.g., at the WHO-AFRO) level (Figure 2). “Integrated” means that each laboratory is connected with a network of laboratory referrals within a tiered system of increasing complexity for laboratory testing (39, 97). For CBL, testing menu examples at each level still need to be made. Of note, this network approach facilitates the standardization of procedures, equipment and consumables, which is most beneficial to CBL given its impact on training, supply, maintenance, quality assurance programmes, and pricing.

### Accreditation and Training

The laboratory networks are coached via a process to achieve accreditation, i.e., the formal recognition of competence. WHO AFRO has established a framework for improving the quality of public health laboratories in LMIC to achieve ISO 15189 standards. This framework, implemented by ASLM, is the Stepwise Laboratory Quality Improvement Process Toward Accreditation (SLIPTA, Box 4) programme. Education and in-service training are provided by innovative contextual teaching tools such as the Strengthening Laboratory Management Toward Accreditation (SLMTA) toolkit. The SLMTA toolkit contains valuable approaches for the clinician-laboratory interphase (e.g., “*Meet the Clinician*” sessions), and its extension with CBL applications has been recommended (27, 41). Notably, compared to clinical chemistry and hematology, CBL is less amenable to standard quality management (due to a lack of quantitative data, multiple parameters, and sampling and transport conditions); therefore, a specific qualitative approach with an emphasis on embedment in clinical care is recommended (27, 92, 138). Options for participating in external quality assessments are, however, rare. An original component—so far performed only in high-income countries—is an internal quality assessment consisting of the “resubmission” of clinical samples (i.e., reregistering and reprocessing) (139).

### Laboratory Infrastructure, Work Force, Metrics, and Connectivity

Laboratory support comprises assistance in terms of infrastructure as well as technical, procedural and management requirements. A sigh emphasis is placed on care for the laboratory workforce, including retention strategies and career paths. However, an unintended downside of creating a laboratory-support sector has been that competent laboratory staff have been attracted away from front-line horizontal health services, where competent CBL staff are frequently limited (98).

Metrics are key to monitoring the implementation process, and monitoring is essential for assessing progress, (re)directing activities and appealing to accountability. This boosts confidence among providers and health authorities and provides arguments for sustained commitment to CBL in a hospital. For microbiological surveillance, the GLASS reports provide an excellent tool for inter-country and longitudinal monitoring (57). Regarding sample transport, Southern and East African countries with high burdens of HIV and tuberculosis have worked out integrated specimen referral logistics, frequently in the context of public-private partnerships, which can be extended to CBL (140)^15^.

### Point-of-Care Diagnostics

Point-of-care diagnostics (POC), or rapid diagnostic testing, have improved the coverage and speed of testing for HIV, tuberculosis and malaria. However, for CBL in LRS, apart from those mentioned above—triage at the first-line, speeding-up of identification and AST of grown cultures—innovations that promote rapid phenotypic AST are not expected in the short-term. Crucially, POC tests do not replace laboratories but are complementary, and their intended use must be well-conceived and validated (43, 98). As an example, the unfocused rolling-out of malaria rapid diagnostic tests resulted in an excess of brands and products with variable accuracies and differences in procedures, hampering harmonization, and user-friendliness (98, 141). Furthermore, the implementation of malaria rapid diagnostic tests resulted in errors along the chains of selection, procurement, procedures, and interpretation as well as manufacturing shortcomings. These findings highlight the need to implement POC in well-established medical and laboratory networks, rather than as stand-alone solutions (142–145).

## Diagnostic Tools for the Clinical Bacteriology Laboratory in Low-Resource Settings

*Scientific literature about diagnostics for AMR is mainly technocentric and driven by needs in high-income markets* *(**24*, *28**)*, *despite the growing markets in LMIC* *(**146**)*. *The present section discusses market challenges and opportunities along the diagnostic pipeline, and a large portion of the information was obtained from the gray literature*.

### Diagnostic Tools for Clinical Bacteriology Compared to Those for HIV, Tuberculosis, and Malaria

CBL face several major limitations in their diagnostics panels, such as the overall low sensitivity and speed of blood cultures and the long turnaround time of AST (27, 62). Molecular methods can partly address these challenges, but in view of accuracy, complexity and cost, they are not yet suitable for most LRS (24, 27). Further challenges for CBL are the specific spectrum of “tropical” pathogens [such as *Salmonella* Typhi (enteric fever) and *Burkholderia pseudomallei* (melioidosis)], the diversity of consumables and the need for “tropicalization”, i.e., protection against heat, humidity and dust (27). Compared to single disease programmes, diagnostics for CBL have been largely neglected over the last several decades. This has resulted in the paucity of LRS-adapted tools currently available or foreseen in the near future (24). Given the lack of harmonization and low volume testing (see below), access and supply constitute additional barriers (27).

### Diagnostic Tools for Clinical Bacteriology: Poor Regulatory Stringency

According to international guidelines (adopted by the WHO) (147)^16^, culture media and stains used in CBL are categorized as “low risk” diagnostics; therefore, regulatory scrutiny is minimal. As an example, pre-market authorization of diagnostics does not require the results of performance testing or quality management audits for manufacturing (147). This means that a diagnostic can be marketed without extended or published information about its performance (precluding comparison with other products), and this also means that significant lot-to-lot variations in performance and stability may occur. This laxity hampers CBL diagnostics compared to those used for malaria, tuberculosis and HIV, which are categorized as “moderate-high” and “high risk,” respectively, and are subject to more stringent regulations (148). In addition, regulations in high-income settings do not cover real-life situations in LRS, such as environmental conditions (temperature, humidity, and dust) and limited end-user education and training. The WHO Prequalification of *in vitro* Diagnostics (149) aims to provide vital technical and procurement support for diagnostics, including product performance testing and manufacturing site inspections. The emphasis is placed on the suitability of diagnostics for the specific environmental and human conditions in LRS, particularly in areas without strong regulatory oversight. However, for the time being, the WHO Prequalification focuses on “priority diseases” such as HIV/AIDS, malaria, hepatitis B and C and does not address diagnostics for CBL.

### Substandard Diagnostics and the Role of Professional Organizations

Substandard and counterfeit (falsified) medical products (“SF medical products”) (147) have rarely been reported for general laboratory diagnostics, although this may, in view of anecdotal but consistent observations, constitute non-recognition and underreporting (29, 150). Customer awareness that is supported by a solid (supra)national legal framework (including vigilance systems and post market surveillance) is pivotal to assess the nature and scale of the problem. The proposed use of unique IVD identifiers and track-and-trace technologies may help to detect SF products in the supply chain (147). Likewise, international professional associations may be “whistle-blowers” for SF products and incite corrective actions from the manufacturer's side, as done by the European Committee on Antimicrobial Susceptibility Testing (EUCAST) for AST disks^17^.

### Diagnostics From Concept to Adoption: Market Interventions

To fuel the diagnostic pipeline, CBL providers and regulatory authorities must align with product developers and manufacturers, especially given the long and costly process involved in the development of diagnostics (27). Market challenges are concentrated around three factors: (i) non-awareness of market needs by manufacturers, (ii) downward pressure on pricing for LMIC diagnostics and (iii) the increased investment needed to generate evidence to support the adoption of new diagnostics (24).

Target Product Profiles (TPP), which list the needs and assets for a given diagnostic in a given setting, are instrumental for communicating diagnostic requirements to developers and manufacturers (24). A preliminary market analysis and a brainstorm session with a multidisciplinary team are conducive to drafting TPP. TPP include intended use, target population, accuracy, time-to-results and cost, as well as items related to deployment in LRS such as the intended end-users, training needs, shelf life and environmental stability (151–153).

The second challenge occurs when commercializing and scaling up the production of diagnostics. Downward pressure on the pricing of diagnostics, as well as production scale-up that is too rapid and lead times that are too short, may impact the manufacturing quality. This has been observed for malaria rapid diagnostic tests (145). By contrast, the standardization of equipment and consumables at the (supra)national level can increase sales volumes and consequently decrease prices (see above). Furthermore, technology transfer in the local production of diagnostics in LRS is a valuable option (154). This is particularly true for CBL, as many stains (like Gram stain) are categorized as “dangerous goods” by the International Air Transport Association (IATA)^18^. For CBL, centralized media production facilities can assure ready-to-use quality-assured culture media at affordable prices and customized volumes (27).

The investment (time, costs, and effort) required for clinical studies of diagnostic performance in real-life situations in LRS is substantial and ever increasing. Therefore, it is perceived as a third challenge by developers and manufacturers. As an example, the U.S.A. Food and Drug Administration-compliant approval of a blood culture system requires a comparative clinical trial with approximately 8,000–9,000 blood cultures (155). Standardized open-access protocols for high-throughput *in vitro* (phase 2) reference testing on contrived samples should be developed to preselect products for clinical studies. Likewise, the biobanking of geographically representative clinical strains from LRS is pivotal to compile representative “tropical” panels for the analytical validation of identification and AST diagnostics. Finally, sample study protocols and evaluation criteria must be publicly available, and diagnostic products may be tested in combined trials at dedicated trial sites (24).

The harmonization of regulation will make the market more attractive to manufacturers. An example is the Pan African Harmonization Working Party on Medical Devices and Diagnostics (PAHWP) (148). The harmonization of regulation facilitates market entry and avoids the duplication of product testing at the country level (148). Furthermore, market shaping can be done by procurement strategies. One of the largest funding agencies of vertical disease programmes, the Global Fund to Fight AIDS, Tuberculosis and Malaria (Global Fund), is making large-volume diagnostic procurements (via a pooled procurement mechanism) based on a combination of price, quality, innovation and the total cost of ownership (i.e., product cost combined with indirect costs such as transport, lot testing, training and quality control). To assure market viability and manufacturer buy-in, the PPM system commits to annual product volumes that allow manufacturers to optimize their production plan and to strengthen their quality management system (156).

### The WHO Model List of Essential *in vitro* Diagnostics

The WHO EDL (see above) provides evidence-based guidance to facilitate the appropriate choice of diagnostics. In line with the already established WHO Essential Medicines List, the WHO EDL is expected to promote access to quality-assured IVDs among health professionals, industry, policy makers, and regulatory authorities. Beyond this, the WHO EDL will provide guidance for the harmonization of diagnostics and external quality assessment programmes and facilitate the group purchasing of diagnostics and orienting (43).

### Diagnostics—The Business Model for the Clinical Bacteriology Laboratory

Surprisingly, little has been published on business cases for CBL in LRS, although knowledge of prices and cost-effectiveness are essential for the acceptance, adoption and sustainability of diagnostics.

At the procurement level, CBL differs from HIV, malaria and tuberculosis. First, the number of consumables (reagents and commodities) needed for CBL is much higher and more diverse, and there is a plethora of manufacturers and suppliers. Second, CBL cannot count on disease-specific international programmes and funders and thus cannot take advantage of large volume procurements and market shaping. Consequently, bulk discounts (28) are not granted for CBL, and prices may be volatile. Furthermore, in other sectors (such as malaria RDTs), it has been observed that, outside of competitive tenders, manufacturers may charge predatory prices for sole source orders (145). Data are rare, but it is estimated that the price for blood cultures in LRS is twice as high as in high-income countries (24).

Regarding price setting for laboratory testing, recent research in the laboratory diagnosis of tuberculosis in India demonstrated a lack of transparency about the breakdown of non-reagent laboratory costs. Furthermore, unwanted practices such as providing incentive fees to referring doctors (an illegal practice in high-income countries) further inflated prices. When asked about price setting for new diagnostic tests, laboratory managers declared that pricing would be set at a 400–700% mark-up on the reagent price, taking into account the patient's willingness to pay (30).

Adding to these challenges is the relatively low cost-effectiveness of CBL. The cost of current antibiotic treatment for bloodstream infections is still relatively inexpensive and consequently does not outweigh the cost of cultures and AST (24). This is in contrast to HIV, malaria and tuberculosis, where even more expensive diagnostics outbalance the costs of treatment (24, 145). Furthermore, the “one who pays” makes a difference in cost-effectiveness (28); in LRS and in the absence of a subsidized projects for CBL (such as the AMR surveillance projects described in Box 5), costs are passed on to the patient and do not necessarily impact hospital management (28). An example of a feasible and cost-effective laboratory strengthening intervention in Ethiopia is shown in Box 6.

Box 5 **Microbiological Surveillance at the hospital level: Examples from Cambodia—DR Congo**

Microbiological surveillance at the hospital level had impact on the local, national, and international (global) level (10). Below are examples of Cambodia and the Democratic Republic of the Congo (DR Congo). Surveillance data were obtained as part of capacity building projects subsidizing consumables for blood culture surveillance.

**Table:** 

EXAMPLE	**Cambodia:** Sihanouk Hospital Center of Hope (SHCH) is a 30-bed non-governmental organization hospital for adults providing healthcare services at a limited cost. In 2016, care was given to 30,500 outpatients and 800 hospitalized patients. Between 2007 and 2016, 22,189 blood cultures were processed yielding 1,888 (8.8%) pathogens (157).	**DR Congo:** Blood culture surveillance network set-up by the National Institute of Biomedical Research (INRB, Kinshasa), with different sampling sites (referral hospitals and two university hospitals) as well as outreach sampling. The main sampling site is Kisantu Hospital in the Kongo-Central Province (275-bed, draining 202.500 persons in 2,400 km^2^)
HOSPITAL AND COMMUNITY LEVEL	Directed antibiotic treatment of invasive infections. The laboratory liaison function is assured by a team of physicians trained in infectious diseases and antibiotic stewardship. Detection and confirmation of melioidosis, a life-threatening caused by *Burkholderia pseudomallei*. Melioidosis was suspected to be endemic in Cambodia but had not been confirmed until blood culture surveillance since 2007 (158). Hospital-based antibiotic treatment guidelines based on cumulative susceptibility data: drafted since 2011 with continuous updates (53). Early detection and investigation of hospital outbreaks e.g., *Burkholderia cepacia* from multidose vials (53). Laboratory staff is active part of in the SHCH infection prevention and control committee.	Directed antibiotic treatment of invasive infections, in particular by demonstrating bacterial co-infections in children with suspected or confirmed severe malaria (159, 160). The inventory of pathogens showed the prominent place of *Salmonella* Typhi among adults and of non-Typhoidal *Salmonella* in children with high resistance rates to first line antibiotics
NATIONAL LEVEL	Coordinated by the Ministry of Health and in collaboration with national and international partners, organization of the first national conference on melioidosis (2010) and the first national workshop on antibiotic resistance (161) and contribution to the Cambodia National Action Plan to Contain Antimicrobial Resistance^10^ and GLASS surveillance. Adding ceftazidime—antibiotic of choice for the treatment of melioidosis—to the 2012 version of the Cambodian Essential Medicines List (162).	Blood culture processing demonstrated non-Typhoidal *Salmonella* as the cause of excess case fatality in outbreaks of *Plasmodium falciparum* malaria (64, 159, 160). The National Malaria Control Programs has referred to this co-occurrence in its 2013 report. Contributing to diagnostic stewardship, in case demonstrating the non-value of the Widal test (antibody test) for the diagnosis of typhoid fever (163). Detection of exceptional drug resistance in *Salmonella* Typhi—i.e., extended-spectrum beta-lactamase (20).
INTERNATIONAL LEVEL	Early alert and monitoring of an outbreak of *Salmonella* Paratyphi A in Phnom Penh (161) complementing unusual increases from *Salmonella* Paratyphi A in international travelers (164–166).	Contributing to global epidemiological mapping and evolution of *Salmonella* Typhi and the non-Typhoidal *Salmonella* (167, 168). Providing data supporting advocacy to vaccination for invasive salmonellosis.

Box 6 **Implementing Clinical Bacteriology in Low Resource Settings–an example of a feasible and cost-effective laboratory strengthening intervention in Ethiopia**

**Setting**: Tikur Anbessa Specialized Hospital (TASH) is an 800-bed public tertiary care hospital in Addis Ababa Ethiopia, considered low resource but moderate infrastructure. The hospital provides referral services for about 200,000 inpatients and 330,000 outpatients annually in Obstetrics and Neonatology, Hematology-Oncology and Complex Surgical care for Adults and Pediatric patients referred from around the country.

**Funding**: The federal government funds hospital operations allocating an annual budget for employee salaries and for running costs, but in addition, patients are charged for some of their medical costs (some antibiotics, laboratory and radiological tests) depending on ability to pay and availability of drugs in pharmacy stocks.

**Context prior to the laboratory-strengthening intervention**:

- Existing bacteriology laboratory with a skeleton staff
- Processing of low volume of specimen (fewer than 2 blood culture specimens per day, despite the complexity of patients treated)
- Problematic quality of results, inconsistent use of Standard Operating Procedures (SOP) and poor relationships with clinicians
- On any given day, 80% of in-patients on medical and pediatric wards and 100% of ICU patients are on broad-spectrum antibiotics
- Less than 1% of inpatients had had microbiological workup

**Example of a minimal bundle for CBL strengthening (adapted from CLSI-QMS01-A guideline)**

- Prioritizing Blood Culture processing to diagnose Blood Stream Infection
- Implementation of 12 core SOPs including standardized identification and susceptibility testing
- Training and supervising of laboratory personnel via consultant visits and existing online training
- Implementation of a practical Quality approach (169).

**Who pays?** For proof of concept, the intervention was funded externally:

- An automated blood culture equipment (BacT/ALERT, value estimated at 150,000 $CAD, donated from bioMérieux)
- First year costs of reagents for processing blood culture specimens for in-patients: 35,000$/year (5,000 blood culture specimens) invested by research funds (RI-MUHC)
- Patients not charged for costs of blood culture testing

**What does success look like?**

- In the first 18 months after implementation, the volume of specimens processed in the CBL increased from <15/day to >75/day
- Standardized identification and susceptibility testing performed for blood cultures and a cumulative antibiogram developed for the institution – demonstrating **exceedingly high rates of *Enterobacteriaceae* were resistant to 3rd generation cephalosporins (>85% resistance) and to carbapenems (>10%)** (170)
- Availability led to recognition of critical importance of CBL, and facilitated first steps toward implementing an antimicrobial stewardship intervention in the institution

**Cost effectiveness is not a straightforward equation**

- Implementation of laboratory laboratory-strengthening intervention can lead to initial increase in antibiotic expenditures when prevalence of drug-resistant infections is high
- **In the first year after implementation of the CBL strengthening intervention**: total antibiotic expenditures (not including antibiotics purchased by inpatients from outside pharmacies) amounted to 9,853,453 Ethiopian Birr (447,885 USD) equivalent to 18% of the total pharmacy budget and significantly higher than the year before; the increase in costs were driven by increased use of meropenem and vancomycin (4 antibiotics accounted for 66% of the total antibiotic budget)
- **After the first year**: A subsequent CBL-supported pharmacist-led antimicrobial stewardship intervention allowed discontinuation or modification of antibiotics in >50% of cases – leading to savings of 1,000,000 Ethiopian Birr (36,000 USD) over 8 months
  **→*Investing in CBL is cost effective provided it is combined with a stewardship intervention and supported by senior administration to ensure sustainability***

The absence of a workable business plan for CBL has undesirable effects. First, high prices for culture media drive CBL to implement the in-house preparation of complex culture media, such as blood culture media, compromising quality and harmonization (24). Moreover, the prohibitive out-of-pocket costs will drive patients and doctors to overtreat with antibiotics and lead to the underuse of the CBL (28). As a result, CBL in LRS are caught in a cycle of low supply and demand. The number of samples processed frequently does not reach the critical volume needed to acquire and maintain quality and experience (27) and further pushes prices upwards while increasing the burden resulting from expired media. The absence of a positive business case is therefore a roadblock to the implementation and sustainability of CBL and AMR surveillance in LRS and needs to be addressed.

### A Role for a Global Alliance for Diagnostics

The sustainability of CBL requires a healthy market and business mechanisms and predictable, long-term financing (171). In a recent series of papers about pathology and laboratory medicine in LMIC, Horton and coworkers highlighted the potential role of global alliances (28). The GAVI Alliance (formerly the Global Alliance for Vaccines and Immunization) is a potential role model^19^. The GAVI alliance is a global health partnership of public and private sector organizations dedicated to “*immunization for all*.” It offers a forum for policy making, advocacy, and strategy and priority setting and strives for in-country ownership and sustainability (e.g., county co-funding and trajectories that promote a gradual reduction of support). A similar global alliance for diagnostics (“*GAMDI*,” which is similar to GAVI but for diagnostics) has been proposed (171) to address market shortcomings and ensure sustainable funding for essential diagnostics with commensurate country commitment. The WHO EDL can be the first step in creating such an alliance (42).

## Workforce: Promote the Expanding Role of the Clinical Bacteriology Laboratory

*The movement of the clinical bacteriology laboratory to the hospital referral level and the expansion of ABS and IPC puts extra strain on the laboratory workforce. It requires broad competence and expertise in the workforce. The section below discusses the preparation of LRS CBL staff for their expanded role. As the literature on CBL training is virtually non-existent, most data are relevant to general laboratory medicine*.

### The Need for a Skilled Workforce at the Clinical Bacteriology Laboratory

The assumption of roles in CBL besides those needed for daily processing of routine patient samples creates new requirements that compound the existing communication gap between clinicians and laboratory staff. Despite the efforts that have been made (e.g., SLMTA trainings, meet-the-clinician sessions), communication between clinicians and laboratory staff is still limited, as laboratory staff are focused on the laboratory work-up and have little few influence on decision-making processes, antibiotic stewardship and infection prevention and control (27, 172, 173). For AMR surveillance, CBL staff face challenges such as the ever changing and difficult-to-understand CLSI guidelines (174, 175) and the complexity of the cumulative antibiogram (115–117). Furthermore, confidently implementing ABS and IPC requires an understanding of healthcare infection dynamics, knowledge of antibiotic treatment, and familiarization with in-hospital nursing and technical processes. Excellent communication and teamwork skills are also valuable assets (122, 124). In high-income countries, these roles are assumed by medical professionals. Their exact titles may vary (e.g., clinical microbiologist, clinical pathologist, and medical microbiologist) as well as their degree of involvement in ABS and IPC, but they are all trained and board-certified to oversee the AMR components (80, 176).

### The Reality Gap: Laboratory Staff in Low-Resource Settings—and How to Turn the Tide

In LRS, CBL are mostly staffed by laboratory technicians (Box 3) (39, 42), who also contribute to other laboratory services and take part in night and weekend shifts. The LRS equivalent of the clinical microbiologist is rare and ill-defined (25), and most CBL are overseen by a manager with little expertise in CBL (27, 92, 177). Human resource management in general laboratory medicine in LMIC is rare (98), and this is particularly the case for CBL (27, 92). Furthermore, there is a high laboratory staff turn-over rate in all African countries (47). Ironically, the revival of laboratory services for HIV, malaria, and tuberculosis (93, 94) has drained skilled staff away from the “horizontal” healthcare system in Sub-Saharan Africa, as does the booming private sector (28).

Furthermore, the term “laboratory staff” and its shorthand term “*lab tech*” cover a myriad of job titles and profiles that differ among countries and are not always consistently defined and certified. Examples are “laboratory technologist” vs. “laboratory technician” (the former being more highly qualified), both of which are used in guidance documents (47). The numerous other cadres involved in CBL are also crucial: phlebotomists, ancillary laboratory workers (auxiliary staff and “lab aides”), information technologists, equipment maintenance staff, quality assurance, and biosafety/biosecurity officers, and cleaning and waste management staff (39, 47). In addition to the gaps between these groups, there are communication mismatches due to the perceived differences in authority between academically qualified clinicians and laboratory staff with basic training (27).

The closing this human resources gap requires broad actions that address job profiles, pre-service and in-service training and active retention plans that are supported by advocacy and regulations within the scope of national laboratory networks and plans (47).

### Job Descriptions and Professional Profiles

In light of the above, the WHO AFRO guidance recommends creating job descriptions for all categories of laboratory staff within the national laboratory network (47). Professional associations (see below) can assume the role of drafting job description templates, which define roles, responsibilities and the qualifications required and list particular competences for all cadres of the CBL staff. Regarding patient care and the processing of samples at the bench, specific CBL requirements apply, for instance, to phlebotomists (blood culture sampling) and biosafety officers (safe destruction of blood cultures) (47). For AMR surveillance, examples of minimum ABS and IPC competencies could include GLASS-compliant data reporting, timely and competent reporting of preliminary culture results and the performance of environmental culture sampling as part of hospital-outbreak investigations. Job descriptions provide a further basis for near-future competence performance and registration (47).

### Pre-service Training: Education and Curricula at Vocational Schools

Although recognized as crucial (47, 93), there is surprisingly little published information about pre-service curricula for laboratory staff. Arneson et al. (178) described their recent experiences in designing and implementing a new medical laboratory curriculum at different universities in East Africa. The starting point was the observation that the superseded curriculum focused on recall rather than on problem-solving competencies. Furthermore, pre-service education shortcomings included the low availability of teaching staff, insufficient skill in the use of electronic teaching media, and the lack of availability of hands-on training facilities and consumables (178). Table 5 lists recommendations for building a curriculum for medical laboratory staff.

**Table 5:** Recommendations for designing and implementing a pre-service curriculum for clinical bacteriology laboratory technicians.

1.	Coordinate with the faculty of the laboratory schools and laboratory supervisors
2.	Base content on job descriptions and responsibilities If not available, work out these job descriptions with relevant stakeholders
3.	Focus on applications, hands-on and problem-solving outcomes
4.	Invest in development of teaching staff, e.g., ∘ objective-writing practices ∘ electronic teaching media (Word, PowerPoint) ∘ hands-on practices
5.	Standardize curriculum and approach, if possible, between institutes
6.	Involve students in active learning
7.	Look for complements with pre-service training

*According to WHO (47) and Arneson et al. (178)*.

### In-service Training, Continuing Education Programmes, and Mentorship Programmes

In-service training primarily focuses on the requirements and qualifications needed for daily tasks in the laboratory. More broadly oriented training can be provided in the scope of continuing education programmes, stand-alone courses and distance learning. In-service training fits into the process of competency assessment and laboratory accreditation. Beyond this, training contributes to personal development, confidence, and leadership—all important for staff retention (39, 47, 93). Furthermore, mentorship programmes and short-term visitor programmes have been promoted and are effective according to the above-mentioned review regarding pathology and laboratory medicine in Africa (98).

### Train All Laboratory Cadres and Train Different Hospital Teams Together

**Figure 3:**
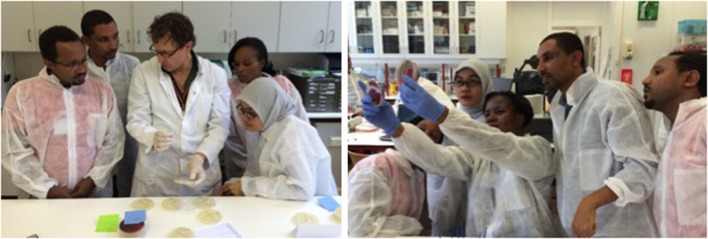
During so-called “*Plate Rounds*,” clinicians and laboratory staff meet in the laboratory and discuss selected cases of infections in a didactic setting. Culture plates including AST results are shown and discussed (e.g., de-escalation of antibiotic treatment (84). Moreover, Plate Rounds can be connected to remote expert advice by telemedicine (180). Originally conceived as a training tool applied in academic medical centers, Plate Rounds provide excellent opportunities for diagnostic and antibiotic stewardship and create liaisons between clinicians and laboratory staff, trainees and in case pharmacists and IPC team (84). The pictures above are “*Plate Rounds*” set-up during the short course “*Hospital-based Interventions to Contain Antibiotic Resistance in Low-resource Settings*” at the Institute of Tropical Medicine. Participants of the microbiology track demonstrate case-based laboratory cultures to their colleagues from the antibiotic stewardship and infection prevention and control tracks (181). Written informed consent was obtained from the individuals for the publication of this image.

All cadres (including phlebotomists and support staff) irrespective of their geographical location should participate in in-service training (47). Whenever possible, “mixed” trainings—i.e., with both clinicians and laboratory staff and that expose laboratory and clinical staff to their counterpart's setting are encouraged. Examples are the “*meet-the-clinician*” sessions in the SLMTA training (39, 177, 179), which should be supplemented with contextual AMR cases. Given the liaison between CBL ABS and IPC, common trainings should also involve the ABS and IPC teams as well as nursing staff—in view of their central role (see above). An interesting experience (developed in high-income countries but obviously attractive to LRS) is the so-called “*plate round*” (Figure 3) (84).

### Laboratory Supervision

To be effective, training must comply with regulations and be monitored with an effective supervisory strategy embedded in the national laboratory health plan (47). Such a strategy includes integrated and harmonized supervisory visits within the tiered laboratory system and mechanisms for feedback and remedial action (47).

### Staff Retention: Remuneration, Workplace Improvement, Training, and Research

Adequate compensation and remuneration are essential for staff retention and motivation (39, 93, 98). Indirect tools for professional recognition also have an impact on existing staff. Such tools include infrastructure renovation and personal development by training (see above) and research (see below) (47, 93). The National Laboratory Strategic Plans developed by countries should outline staff requirements (numbers, qualifications, and their distribution) with an emphasis on under-served areas and identify adequate financial resources (47).

African experts promote research as a valuable tool to strengthen national health laboratory network systems (47, 93). Research can be conducted at all levels of the network but should be prioritized and coordinated by a National Research Plan. Apart from generating information vital for developing and implementing CBL (27) and guiding public health measures, research is beneficial for staff capacity building (93). AMR surveillance and research regarding ABS and IPC are therefore opportunities for the CBL. However, a shortage of academically trained laboratory scientists, as well as a lack of career advancement opportunities for them in many LRS institutions, may hinder the exploitation of these opportunities (182, 183).

### Advocacy and the Role of Professional Associations

The paucity of published literature about pre-service training and the unclear professional profile of laboratory staff in LRS (25) are in part due to the low representation by professional CBL associations. Professional associations provide information and dedicated training, legal advocacy, mentoring and career guidance to professionals (184). They can generate job descriptions and competency assessments and regulate the certification and registration of professionals (47). The representation of laboratory professionals at decision-making levels and in in-country medical laboratory councils is needed (93). As is the case for pathologists, clinical microbiologists in LRS should be more visible in their professional life and contacts with the public health authorities, students, and early career professionals (28). African institutes (ASLM and the African Union) obviously can play a pioneering role in this regard, and counterpart professional associations in high-income countries can facilitate this (28). The WHO recently published a comprehensive document about biomedical engineers (184), which was highly instrumental for future professionals and stakeholders (vocational schools, health authorities and industry). Similar analyses would be highly useful for CBL and other laboratory staff.

## Other Challenges for Clinical Bacteriology Laboratories at the Hospital Level in Low-Resource Settings

*The paragraph below briefly discusses other challenges and advantages for the clinical bacteriology laboratory at the hospital level in low-resource settings. In such conditions, selecting feasible targets and developing creative solutions is key. Priorities for research and action are highlighted*.

### Size Matters

Most hospitals in Sub-Saharan Africa are small (185). This not only puts a strain on the CBL staff but also means that staff that are competent in ABS and IPC are rarely present (81). However, community hospitals in the USA and Australia, when facing similar problems, also demonstrated the advantages of being small in size via the encouragement of personal interactions and a culture of collaboration, centralized management and support, and flat governance structures that stimulated ownership and commitment—all of which are pivotal for successful CBL, IPC, and ABS (186–189). Furthermore, task shifting may be considered; in the absence of qualified microbiologists, trained clinicians may take up the role of liaison with the CBL (Box 3) (92), and trainees and pharmacy technicians may take on daily tasks involved in ABS (189). In addition, telemicrobiology may facilitate connections with experts (190), although regulations have to be created and conditions favoring adherence and sustainability need to be studied (186).

### Remote and Rural: Sample and Data Transfer

Connectivity is key for referral hospitals located in rural and remote settings. Detailed information on transport conditions and optimal delays for the transfer of clinical samples from first-line referral centres can be found in published guidelines (69). Blood culture bottles can be used for bed-side inoculation and the collection of cerebrospinal fluid, pleural exudates, and joint fluids (69, 191). Flocked and foam swabs may be attractive for the transport of specimens or isolates (192, 193), but they require the validation of their performance in tropical conditions beyond the CLSI M40-A2 standard (194, 195). Regarding sample transport, efforts, and research should not only address hardware tools such as dedicated containers (196) and drones (197) but also software used for data tracking, result reporting and logistics (198, 199). Business models for transportation can be co-organized with vertical programmes or with the private sector; in Burkina Faso, ASLM worked out an integrated specimen referral system for respiratory diseases via a public-private partnership with the national postal system (140).

### Biobanking and Confirmatory Testing

The GLASS manuals (200, 201) highlight the importance of raw data verification and confirmatory testing in cases of unusual and clinically or epidemiologically alarming AMR profiles. CBL in hospital laboratories could be alerted by “flags” that would prompt them to send the bacteria to the National Reference Laboratory for confirmation. Based on the experience of the authors, the impulse to store and ship the bacteria may occur too late for timely action, in particular when CBL staff are not acquainted with the expert rules. As part of diagnostic stewardship (Box 3), CBL should therefore define which specimens or culture plates to “keep on the bench” for review or follow-up testing. Furthermore, the raw data recording system (either paper-based or digital) should allow sufficient detail to trace clerical or procedural errors. The WHO “Basic laboratory procedures in clinical bacteriology” describes simple procedures for the short-term storage of bacteria that are feasible in a basically equipped laboratory (202–204). Beyond patient-centred and surveillance purposes, storing bacterial strains (and/or clinical specimens) and combining these with extensive clinical and demographic data in biobanks are essential not only for research purpose but also for the development of diagnostic tools adapted for use in LRS. However, developing biobanks in LRS requires strengthening of biological resource management infrastructures (205). In this perspective, the International Agency for Research on Cancer (IARC) has recently launched the Biobank Learning platform, a freely accessible resource providing learning and training material for professionals involved in biobank-based research in LMICs (206).

### Biosafety, Biosecurity, Waste Management, and Environmental Impact

Another consequence of having CBL at district level hospitals is the need for waste management and the regulation of environmental impacts (39, 47). Culture-amplified blood culture specimens and agar plates contain billions of bacteria requiring safe destruction—preferably by autoclaving—before incineration (207). Among them, there are *Salmonella* Typhi, *Burkholderia pseudomallei*, and *Brucella* spp., which are categorized according to the European Union Directive 2000/54/EC (169) as belonging to risk group 3 and therefore requiring stringent biosafety measures. They are also categorized as Category B Bioterrorism Agents (208) requiring enhanced disease surveillance and biosecurity measures (209). During the preparation of the next version of the WHO Laboratory Biosafety Manual (207), the concepts of risk management and sustainable technology will be considered along with the use of measures proportionate to the actual risks in a tiered system (210).

## Conclusion

The placement and consolidation of the clinical bacteriology laboratory at the hospital-referral level in low-resource settings facilitates the exploitation of routine patient care data for surveillance, antibiotic stewardship and infection prevention and control. This involves a synergistic tripartite effort at the frontline of the containment of the emergence and spread of multidrug resistant bacteria (211). A major leap forward can be achieved if challenges related to staff, funding, scale and the specific nature of clinical bacteriology are prioritized. Much can be learned from the past decade of laboratory improvements spurred by vertical programmes for HIV, tuberculosis and malaria. The mobilization of resources coordinated by countrywide national laboratory plans and interventions tailored by a good understanding of hospital microcosms will be key to success. This will be further supported by much-needed market interventions and business models for diagnostics. The future clinical bacteriology laboratory in low-resource settings will not be an “entry-level version” of its counterpart in high-resource settings, but a purpose-built, well-conceived, cost-effective and efficient laboratory ready to assume its place at the frontline of antimicrobial resistance containment.

## Author Contributions

JJ did the literature review, writing of the initial draft and revisions, figure design, and project administration. LH contributed to literature review, revisions, and provided critical review. MS, OL, TP, DA, CY, and OV provided critical review and commentaries. JJ and OV had the rationale for this work, contributed to literature review, and supervised manuscript revisions. LH additionally contributed to figure design.

### Conflict of Interest

The authors declare that the research was conducted in the absence of any commercial or financial relationships that could be construed as a potential conflict of interest.

## Footnotes

^1^World Bank. Available online at: https://data.worldbank.org/country

^2^http://apps.who.int/iris/bitstream/handle/10665/193736/9789241509763_eng.pdf?sequence=1

^3^http://apps.who.int/gb/ebwha/pdf_files/WHA68/A68_20-en.pdf

^4^Center for Disease Dynamics Economics and Policy. *State of the World's Antibiotics*. CDDEP: Washington, DC (2015).

^5^*The Global Health Security Agenda*. Available online at: https://www.ghsagenda.org/

^6^Copenhagen WHO Regional Office for Europe. Central Asian and Eastern European Surveillance of Antimicrobial Resistance (CAESAR).

^7^Liverpool School of Tropical Medicine. *Supporting Surveillance Capacity for Antimicrobial Resistance: Regional Networks and Educational Resources Summary Observations Concerning these AMR networks*.

^8^*WHONET Software and Educational Tutorials*. Available online at: https://www.who.int/medicines/areas/rational_use/AMR_WHONET_SOFTWARE/en/

^9^*GLASS Country Participation*. Available online at: https://www.who.int/glass/country-participation/en/

^10^Cambodian national policy to combat antimicrobial resistance.

^11^*EUCAST Methodology RAST*. Available online at: http://www.eucast.org/rapid_ast_in_blood_cultures/

^12^CDC2003, updated (2017).

^13^*WHO WASH Infosheet*. Available online at: https://www.who.int/water_sanitation_health/facilities/amr-ipc-wash-flyer-nov16.pdf?ua=1

^14^ASLM. Available online at: http://www.aslm.org/

^15^GLI. *GLI Guide to TB Specimen Referral Systems and Integrated Networks*. Available online at: www.stoptb.org/wg/gli

^16^IMDRF. Available online at: http://www.imdrf.org/

^17^EUCAST AST. Available online at: http://www.eucast.org/ast_of_bacteria/warnings/

^18^IATA. Available online at: https://www.iata.org/publications/dgr/Pages/index.aspx

^19^GAVI. Available online at: https://www.gavi.org/
